# High-resolution comparative mapping among man, cattle and mouse suggests a role for repeat sequences in mammalian genome evolution

**DOI:** 10.1186/1471-2164-7-194

**Published:** 2006-08-01

**Authors:** Laurent Schibler, Anne Roig, Marie-Françoise Mahe, Pascal Laurent, Hélène Hayes, François Rodolphe, Edmond P Cribiu

**Affiliations:** 1 Laboratoire de Génétique biochimique et de Cytogénétique, Département de Génétique Animale, Institut National de la Recherche Agronomique (INRA), Centre de Recherche de Jouy, 78352 Jouy-en-Josas, Cedex, France; 2 Mathématique, informatique et génome, Institut National de la Recherche Agronomique (INRA), Centre de Recherche de Jouy, 78352 Jouy-en-Josas Cedex, France

## Abstract

**Background:**

Comparative mapping provides new insights into the evolutionary history of genomes. In particular, recent studies in mammals have suggested a role for segmental duplication in genome evolution. In some species such as Drosophila or maize, transposable elements (TEs) have been shown to be involved in chromosomal rearrangements. In this work, we have explored the presence of interspersed repeats in regions of chromosomal rearrangements, using an updated high-resolution integrated comparative map among cattle, man and mouse.

**Results:**

The bovine, human and mouse comparative autosomal map has been constructed using data from bovine genetic and physical maps and from FISH-mapping studies. We confirm most previous results but also reveal some discrepancies. A total of 211 conserved segments have been identified between cattle and man, of which 33 are new segments and 72 correspond to extended, previously known segments. The resulting map covers 91% and 90% of the human and bovine genomes, respectively. Analysis of breakpoint regions revealed a high density of species-specific interspersed repeats in the human and mouse genomes.

**Conclusion:**

Analysis of the breakpoint regions has revealed specific repeat density patterns, suggesting that TEs may have played a significant role in chromosome evolution and genome plasticity. However, we cannot rule out that repeats and breakpoints accumulate independently in the few same regions where modifications are better tolerated. Likewise, we cannot ascertain whether increased TE density is the cause or the consequence of chromosome rearrangements. Nevertheless, the identification of high density repeat clusters combined with a well-documented repeat phylogeny should highlight probable breakpoints, and permit their precise dating. Combining new statistical models taking the present information into account should help reconstruct ancestral karyotypes.

## Background

Comparative mapping represents a major approach in providing new insights into dynamics of genome evolution. Since the pioneer studies that showed linkage conservation among genomes [1,2], genome comparisons have been carried out in about 30 mammalian species [3]. Although Fluorescent In Situ Hybridization (FISH) and painting analyses have contributed significantly to the identification of conserved syntenies among species [4], the number of ordered gene maps is still too small to draw fully meaningful inferences on chromosomal evolution. In cattle and goats, we had previously reported [5] a high level of intra-chromosomal rearrangements and the existence of preferential breakpoints over the whole genome, which have been confirmed by Radiation Hybrid (RH) mapping data [6-19]. Recently, two high-resolution human-bovine comparative maps have been reported, based on data from the human sequence and bovine RH panels [6,7]. These studies have improved the genome-wide comparative coverage by ~20% between man and cattle and identified 195 and 161 segments with conserved gene order, respectively. The availability of whole genome sequences for man, mouse and rat and in draft format for eight other mammalian species has made it possible to evaluate the rates of chromosomal evolution and to detect segmental duplication in most of the primate-specific breakpoint regions [8]. In addition, Everts-van der Wind et al [9] have analyzed the evolution of centromere and telomere positions and the gene content within evolutionary breakpoint regions in cattle versus man.

Here, we have explored the relationships between chromosomal rearrangements and the density of interspersed repeats, since these represent a prevalent feature of mammalian genomes. Indeed, all mammalian genomes present essentially the same four classes of transposable elements (TEs): autonomous long interspersed nucleotide elements (LINEs), LINE-dependent RNA-derived short interspersed nucleotide elements (SINEs), retrovirus-like elements with long terminal repeats (LTRs such as endogenous retroviruses ERVs and MaLRs) and DNA transposons (see [10-13] for review). The age and history of these repeats have been inferred from phylogenic analyses (for review see [14,15]), suggesting that most mammalian TEs are related and thus can be divided into lineage-specific repeats (inserted after the divergence of the studied species) and ancestral repeats (already present in a common ancestor). Moreover, TEs can undergo broad bursts of amplification in a lineage specific way, potentially leading to speciation as suggested in Primates [16].

In order to carry out this study, first we have produced an updated version of the bovine physical map constructed in our laboratory [17], and have used it to extend and refine the bovine comparative map with man and mouse. This has led to a high resolution comparative map integrated with the most recent bovine genetic map [18] and FISH data [19]. Second, based on this comparative map, we have identified and examined the intervals between segments of conserved gene order (evolutionary breakpoint regions) among the cattle, human and mouse genomes. We have found that these breakpoint regions are enriched in lineage specific repeats: Alu repeats in man and SINEs in mouse. Since previous studies have suggested that TEs could be involved in chromosomal rearrangements, we propose that the increased density of TEs and especially SINEs in evolutionary breakpoint regions has a role in mammalian chromosome evolution and we discuss the recent findings of Murphy *et al *[8] on the implication of segmental duplication.

## Results

### Updated bovine physical map

Our first autosomal bovine physical map reported in 2004 [17] comprised 6615 contigs of which 747 were anchored to the cattle genome. In the present work, we have anchored 350 additional contigs by PCR-screening of 400 new well-distributed microsatellites. Moreover, we have produced 53,550 BAC end sequences (BES) from 26,935 INRA BAC clones in collaboration with the Genoscope and collected 28,468 BES from the CHORI-240 library via Genbank. BLAST analyses of these BES produced 38,469 valid hits, of which 27,676 correspond to known or predicted human genes and 10,793 are located outside genes. BLAST statistics are similar in all cases, i.e. 88% mean identity percentage, 1.6 10^-07 ^mean e-value and 190 bp mean homology size. As a result, 9,323 INRA and 4,318 CHORI-240 bovine clones have been anchored to the human sequence.

To date, the updated version of our physical map comprises 5,081 contigs ranging from 150 kb to 5.5 Mb in size (600 kb on average). Among these, 860 are anchored to the bovine genome by 1,763 markers, 3,444 to the human sequence and 774 to the genome of both species. These 774 contigs comprise 1121 bovine markers and 2,127 hits on the human genome. Data are available through the cattle webFPC [20] or the BovMap database [21].

### An integrated high-resolution comparative autosomal map for cattle

To construct this comparative autosomal map, first we used the 774 contigs anchored to both the bovine and human genomes. Then, we added 598 FISH localizations (131 microsatellites and 467 genes from [19], 213 of which are also mapped to the contigs). Since contigs contain several BES, they are anchored to human genome with 4290 positive BLASTs. Although the Btau 2.0 whole genome assembly was released in the course of this work, it was not used to draw meaningful conclusions for the following reasons: (1) blasting large marker sets, which had not been used to build Btau 2.0 assembly, showed that it contained partial and sometimes erroneous data (markers detected in more than one scaffold or at several positions in the same scaffold and markers from different bovine chromosomes present in the same scaffolds) and (2) since the Btau 2.0 scaffolds were built based mainly on syntenic relations with the human genome, there may be a bias towards a reduced number of rearrangements between these two species. Thus, the current bovine assembly should be exploited with care and we only used it to anchor 1519 additional microsatellites to the human sequence.

As a result, our comparative map comprises 2721 loci anchored to both the bovine and human genomes [see additional file 1 and additional file 2].

According to the bovine genetic map, our physical map covers 90% of the bovine autosomes and only BTA21 has a coverage below 80%. Bovine orthologous regions have been identified for about 91% of the human autosomes, leaving 166 human segments (~2 Mb on average) without comparative mapping information.

Our results reveal 68 conserved synteny segments between bovine and human autosomes, in good agreement with previous studies [6,19]. If gene order is taken into account, we identify 211 segments, of which 198 include at least two markers. Thus, our work has extended or refined 72 known segments with conserved gene order and identified 33 new segments. Only nine previously reported segments based on single FISH localizations have not been confirmed or remain inaccurate in the present study (gene names and cytogenetic locations indicated in red in additional file 1: PPCB, NAB1, BMPR1B, TXK, GM2A, GALT, PIGR, PIM1, ACPP).

### Analysis of evolutionary breakpoints

**Figure 1:**
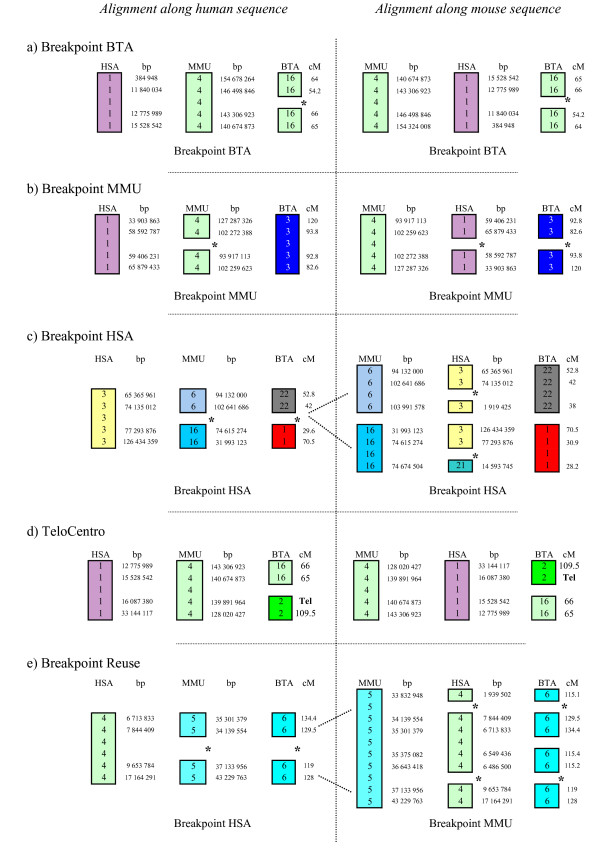
**Principle of the classification of breakpoints**. Classification of breakpoints is exemplified by real data. Chromosomes for each species are drawn as colored boxes, with chromosome numbers indicated in the boxes. Positions along the genome (base pairs or cM) are written on the right. Breaks in boxes and * indicate evolutionary breakpoints. Genomes are drawn aligned along the human and mouse sequences. a) The breakpoint occurs solely in the bovine genome, in both genome alignments. This situation is classified as a BTA breakpoint. b) The breakpoint involves two mouse chromosome segments based on the human alignment. When aligned on the mouse genome, these segments define breakpoints in both human and bovine genomes. This situation is classified as a MMU breakpoint. c) The breakpoint involves both the bovine and the mouse genomes based on the human alignment. When aligned along the mouse genome, these regions define breakpoints only in the human genome. This situation is classified as a HSA breakpoint. d) The breakpoint involves either telomeres or centromeres. This situation is classified as TeloCentro. e) The breakpoint is classified in a different way according to the human and mouse alignments. This situation is classified as a breakpoint Reuse.

Evolutionary breakpoint regions between two species are defined as the interval between two segments of conserved gene order. They are classified into four types, based on their likely origin (see Material and Methods and Figure 1). Repeat densities have been computed for each class of repeats and type of breakpoints and compared to the densities along the human or mouse chromosomes. Repeat densities were not computed along the bovine genome because of flaws in the bovine genome sequence. Indeed, bovine scaffolds are in general smaller than the corresponding BAC contig lengths and contain many large gaps. Scaffolds contain only one third to one half of the sequence of the corresponding genomic region. This is consistent with assembly statistics describing that ~34% of the bovine genome are covered by anchored scaffolds. Moreover, typical errors in draft genome sequences include misassemblies of repeated sequences, collapses of repeated regions, making such sequences difficult to use for repeat content analysis.

**Figure 2:**
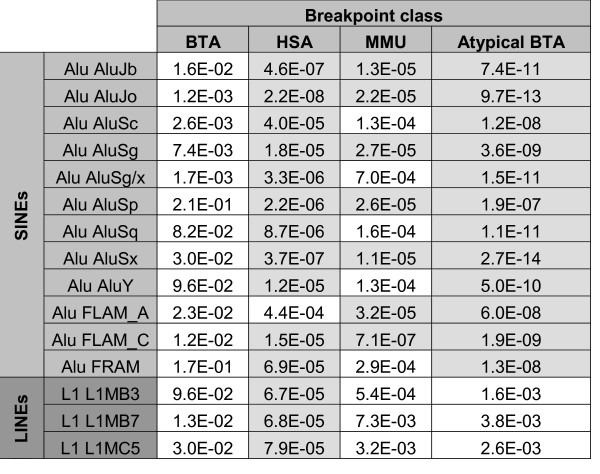
**Results of the one-sample Kolmogorov-Smirnov test in the human sequence**. P-values for the "greater" hypothesis are shown for each class of breakpoints and for repeats with significant results. Highly significant p-values are indicated with a light grey background.

**Figure 3:**
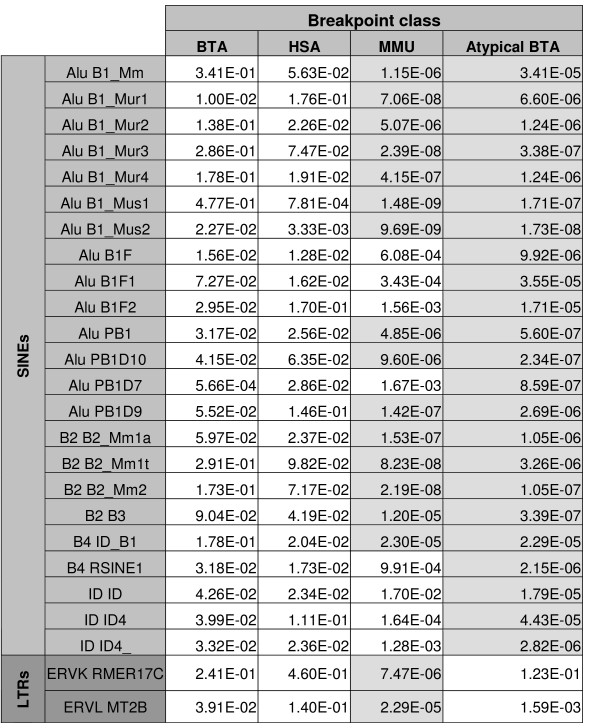
**Results of the one-sample Kolmogorov-Smirnov test in the mouse sequence**. P-values for the "greater" hypothesis are shown for each class of breakpoints and for repeats with significant results. Highly significant p-values are indicated with a light grey background.

The one-sample Kolmogorov-Smirnov test, based on the repeat density, showed an increased density of several interspersed sequences in certain regions. P-values are given in Figure 2 and Figure 3 [see also Additional file 3].

**Figure 4:**
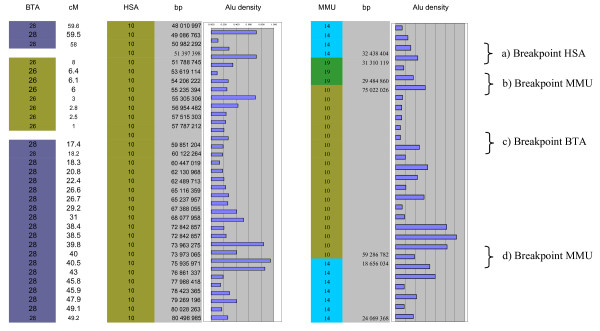
**Examples of increased TE density**. The figure presents the comparative bovine/human/mouse map for a segment of HSA10. a) Alu density is increased in the human genome in the vicinity of an HSA breakpoint. b) Alu density is increased in the mouse genome, in the vicinity of a MMU breakpoint. c) No repeat density increase is observed in the human or mouse genome in the vicinity of a BTA breakpoint region. d) Alu density is increased in both human and mouse genomes in a MMU breakpoint, probably due to the strong correlation observed between the local SINE density in mouse and the Alu density in orthologous loci in man.

**Figure 5:**
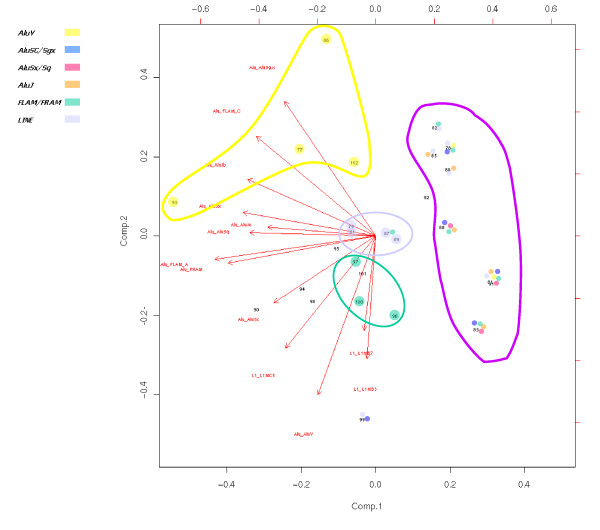
**Principal Components Analysis of human breakpoint repeat content**. Breakpoint region numbers are plotted against the two major component axes, arrows indicating the contribution of each repeat. High enrichment in repeats is indicated for each breakpoint by colored spots.

We have detected a highly significant increased density of SINE Alu repeats in the human sequences corresponding to HSA and MMU breakpoints but not to the BTA breakpoints (see Figure 4 for an example). A Principal Components Analysis (Figure 5) suggests that most of the HSA breakpoints are enriched in various repeats: three HSA breakpoints contain only a high density of ancient Alu repeats, four only a high density of young Alu repeats and four display an elevated frequency in L1 repeats (L1MB3, L1MB7 and L1MC5).

The same analysis performed on MMU breakpoints shows a strong correlation between all repeat densities (data not shown). Analysis of the mouse sequences reveals an increased density of some rodent specific SINEs B1, B2, B4 and ID in MMU breakpoints, whereas the BTA and HSA breakpoints do not show any difference with the whole mouse genome. Moreover, 23 MMU breakpoints are enriched only in ERV-L (MT2A, MT2B B or RMER10) or ERV-K RMER17C. Five of these MMU breakpoints also delineate breakpoint regions in rat, suggesting that they occurred in the *Mus *lineage.

Human sequences of 28 BTA breakpoints display an Alu repeat content typical of HSA breakpoints, with an increase mainly in the oldest Alu repeats (FRAM, FLAM, AluJ, AluSx). Likewise, a repeat content typical from MMU breakpoints is also observed in the orthologous mouse sequences.

Moreover, a search for centromeric or telomeric satellites in the human sequence has shown that 24 breakpoint regions contain at least two satellite repeats. About 71% of these breakpoints involve two different chromosomes and may thus be considered as interchromosomal breakpoint regions, whereas 29% may be intrachromosomal breakpoint regions [see additional file 3]. Although not statistically significant, satellites may be slightly over-represented in interchromosomal breakpoints. About 11% and 4% of interchromosomal and intrachromosomal breakpoint regions contain satellite repeats, respectively.

## Discussion

### Discrepancies between different bovine comparative maps

The autosomal comparative map reported here is in good agreement with previous studies based either on FISH or RH mapping of genes. Most discrepancies are due to differences between framework maps, especially in that reported in 2004 by Everts-van der Wind *et al*, which was built with a low resolution RH_5000 _panel and a reduced number of backbone microsatellites. Erroneous identification of orthologous sequences may also introduce some errors in all studies and contribute to the observed discrepancies. Moreover nine cattle FISH localizations have been found to be inconsistent, probably due to false orthologous gene pairs or mapping errors.

### Breakpoint regions display a high density of lineage specific repeats

We have shown an association between the density of interspersed repeats and the evolutionary breakpoints in the human and mouse genomes. Interestingly, the repeat increase in these regions concerns only repeats having spread after the divergence of these species. Moreover, for a given species, only breakpoint regions having occurred within this species are enriched in repeats and more surprisingly, in species specific repeats, except for MMU breakpoints that appear to be Alu rich in the human sequence. This latter result agrees with the strong correlation observed between the local SINE density in mouse and the Alu density in orthologous loci in man [15]. Common biological factors governing the insertion and/or retention of repeats in particular regions or the phylogeny of SINE repeats (Alu and B1 repeats derive from a common ancestor 7SL RNA) could explain these correlations.

Conversely, human sequences spanning regions of BTA breakpoints do not reveal any enrichment in Alu repeats, which suggests that they may be associated with repeats having different insertional characteristics. Bovine lineage specific SINEs or LINEs Bov-tA, Bov-A2 or Bov-B could be good candidates for such repeats and this hypothesis should be assessed after completion of the bovine whole genome sequence.

Moreover, 28 BTA breakpoints show a typical HSA and MMU repeat content in the corresponding human and mouse sequences. If we assume that Rodents diverged from a Primate-Artiodactyl clade, about 50% of the human rearrangements should have also occurred independently in mice, suggesting a very high frequency of convergent evolution. If one rejects this hypothesis, then Artiodactyls may be more distant from human than are rodents. Thus, this analysis of the repeat content in evolutionary breakpoint regions supports the recent hypothesis that Artiodactyls diverged from a Primate-Rodent clade [22-24].

Since evolutionary breakpoints appear to be enriched in specific interspersed repeats, widespread activity of TEs could be involved in genome evolution. Indeed, they provide material for DNA mispairing and thus can lead to genomic instability and rearrangements. A key role of TEs in intrachromosomal rearrangements (large deletions, translocations, inversions) has been evidenced in *Drosophila *[25-31], Mosquito [32-34], Man [32-34] and Maize [35]. Likewise, in human tumors, the high density of repetitive DNA in a given region provides hot spots for homologous recombination and mediates translocation processes [36].

Moreover, Alu mispairing could have generated many segmental duplications [37,38] that are found in most primate-specific breakpoint regions, suggesting their role in chromosomal rearrangements [8,39-41].

However, it is possible that repeats and breakpoints accumulate independently but in the same region where modifications are better tolerated. High repeat densities could also be a consequence of breakpoints as suggested by Dobigny *et *al [42] i.e. major regulation pathway of repatterned genomes could be transiently inefficient, thus resulting in conditions suitable for TE amplification.

### An overall model of genome evolution

Genomes may evolve cyclically under the influence of three main factors: transposon normal activity, bursts of transposon activity and dynamics of centromeres and telomeres.

(i) Normal transposon activity may be responsible for most small intrachromosomal rearrangements (small duplications, deletions or inversions... for review, see [43]).

(ii) Bursts of transposon activity result in a dramatic increase of the local repeat density and thus, may induce large intrachromosomal rearrangements directly or indirectly by segmental duplications. For example, Alu repeats and rodent SINEs could have given rise to most of the intrachromosomal rearrangements during a middle term period (80–50 Million years (Myr)), while during a more recent period (4–10 Myr), ERVs could have caused at least 18 rearrangements in the ancestor of the Muridae and five in the mouse.

(iii) Chromosome fusions or fissions may have produced most interchromosomal rearrangements, as suggested by the proportion of interchromosomal breakpoints containing centromeric and telomeric satellites (present study) and by early cytogenetic analyses [44]. Likewise, a recent study has revealed a clustering of centromere and telomere sequences at the site of evolutionary breakages [8]. Telomeres may have mediated or enhanced these rearrangements, as demonstrated for subtelomeres [45]. It has been shown that centromeres can be repositioned or created *de novo *and thus they could be involved in stabilizing interchromosomal rearrangements.

This model agrees both with the random breakage and the 'fragile breakage' models proposed by Nadeau [2] and Pevzner [46,47], respectively. On the one hand, rearrangements may occur in repeat-rich regions that are roughly randomly distributed along the genome, even if SINEs are preferentially located in GC-rich regions whereas LINEs are essentially present in AT-rich regions. On the other hand, the high density of repeats could promote both hot spots of chromosomal evolution [48] and segmental duplications, in accordance with the fragile breakpoint model. Moreover, the mammalian genome tolerates insertion and accumulation of repeats only in a limited number of regions as indicated by the clustering of some repeats in orthologous regions from different species. Repeat families may have sequentially gathered in the same regions, potentially leading to successive rearrangements. Such regions may thus appear as 'fragile' and could explain that nearly 20% of the breakpoints are reused during mammalian evolution [8]. In addition, some regions could be involved in evolutionary breakpoints in one species and could lead to disease in another, as suggested by the close relationship between evolutionary breakpoints and neoplasia-associated human chromosome sites [5].

## Conclusion

In this paper, we report a high-resolution integrated bovine comparative map with man and mouse based on microsatellites, INRA bovine contigs, BAC End sequencing and previous FISH data. This comparative physical map should be useful for the assembly of the bovine whole genome sequence.

Moreover, it has allowed us to analyze the DNA content in breakpoint regions to reveal specific repeat density patterns. Thus, 'junk DNA' may have played a significant role in chromosome evolution and genome plasticity. Periodic phases of intrachromosomal rearrangements (due to interspersed repeats), followed by interchromosomal rearrangements (providing new opportunities for later intrachromosomal recombinations) could be the driving force of genome evolution. Interestingly, amplification of TEs has been described as a response to stress factors either of environmental origin or genomic nature (see [49] for review). In our model, TE mediated chromosome evolution, leading to new gene regulations and expressions, could thus represent an inventive mechanism to speed up fitness to new environmental conditions, leading to speciation.

However, more studies are required to ascertain whether increased TE density is the cause or the consequence of chromosome rearrangements. Moreover, we cannot rule out that repeats and breakpoints accumulate independently in the few same regions where modifications are better tolerated. Nevertheless, high density repeat clusters combined with a well-documented repeat phylogeny should highlight probable breakpoints, and permit their precise dating. New statistical models of evolution should be developed, taking into account not only the parsimony principle but also the present information in order to help reconstruct ancestral karyotypes.

## Methods

### Anchoring the physical map

The INRA bovine BAC library was screened by PCR using published microsatellite primer pairs (see BovMap database [21]) as previously described [17].

In collaboration with the Genoscope, we produced 53,550 BES from 26,935 INRA BAC clones and we collected another 28,468 BES from the CHORI-240 library via Genbank. Similarities with the human sequence (NCBI built 35) were searched for by BLASTN after repeatmasking ([50]) We used an expectation value E of 10^-5 ^as the significance threshold for comparisons, since this value was shown to provide 95% accuracy in identifying orthologs [51]. Coordinates in the human sequence were then obtained for each retained hit using GoldenPath [52]. After localizing microsatellites in the bovine scaffolds by BLASTN analysis, the position of the bovine scaffolds on the human sequence were determined using BLASTN with parameters set to optimize detection of distant homologies (Ensembl BLASTView: W9, M1, N-1, Q2, R1).

### Segment identification and integration of FISH and bibliographic data

After aligning the contigs along the bovine genome using the Ihara and USDA97 linkage maps [18,53], microsatellite BLAST results and FISH-mapping data were integrated into this framework map.

The boundaries of conserved segments between the bovine and human genomes were defined according to a parsimonious interpretation. Discrepancies in microsatellite and FISH-mapped gene locations between backbone maps were resolved by choosing the location that minimized rearrangements between the bovine and the human genomes.

### Searching for particular sequence features in breakpoint regions

Evolutionary breakpoint regions between two species were defined as the interval between two segments of conserved gene order. As illustrated in the left part of Figure 1, breakpoint regions were classified into four categories, based on the human alignment:

- BTA breakpoints, having probably taken place in the bovine lineage, when a perfect co-linearity is observed between human and mouse maps, but not with the bovine map. (Figure 1a: the breakpoint region is identified only in cattle).

- MMU breakpoints, having probably taken place in the mouse lineage, when a perfect co-linearity is observed between human and bovine maps, but not with the mouse map (Figure 1b: the breakpoint region is detected only in mouse).

- HSA breakpoints, having probably taken place in the human lineage, when no co-linearity is observed among human, mouse or bovine maps. (Figure 1c: the breakpoint region is identified both in cattle and mouse).

- TeloCentro, when either telomeres or centromeres are involved in the breakpoint (Figure 1d).

The same classification was also performed based on the mouse alignment (right part of Figure 1). When identical breakpoint regions fell into different categories according to the human or mouse alignments (Figure 1e), they were discarded from the global analysis because they could represent regions of breakpoint reuse. Breakpoints involving centromeres or telomeres were likewise discarded because these regions may be poorly represented in the genome assemblies and rearrangements may have been mediated by centromeres or telomeres, independently of the repeats we wanted to analyze.

Human and mouse chromosome masked sequences (chromFaMasked.zip) and repeat annotations (chromOut.zip) were downloaded from UCSC [54].

The density (number of repeat per Mb of DNA) of each class of repeats was computed for each kind of breakpoint region. Likewise, densities were also computed along each chromosome in sliding windows of different sizes (0.5, 1, 2, 3, 4 and 5 Mb). The proportion of densities, in these sliding windows, greater than the density in each repeat region was then computed for each class of repeats. These calculations were performed using sliding widows of a size range similar to the breakpoint region size.

Enrichment was tested by applying the one-sample Kolmogorov-Smirnov test to these proportions (distribution under the null-hypothesis is uniform on [0,1]) and using the R software.

Classification results, positions on human and mouse sequences, repeat densities and proportions of frequencies are available as supplementary data (break_supp).

## Authors' contributions

L. Schibler conceived the study, participated in its design and coordination, built the bovine physical map, carried out bioinformatic and statistical analyses and drafted the manuscript. A. Roig, MF. Mahe and P. Laurent performed the BAC library screening. F. Rodolphe helped to design the statistical analysis and to draft the manuscript. H. Hayes kindly provided FISH data and participated in the revision of the manuscript. EP. Cribiu contributed to the preparation and revision of the manuscript and gave final approval of the version to be submitted.

## Supplementary Material

Additional File 1 **comparative_supp**. This excel file contains the comparative maps aligned along each genome in three different pages and gives details on their genome coverage. Click here for file

Additional File 2 **Cattlemap_supp**. This file contains a graphical version of the bovine map. Click here for file

Additional File 3 **breakpoint_supp**. break_supp.xls details breakpoint analysis results, including breakpoint regions description, proportions of densities observed above the density of each class of repeats.... Click here for file
