# Supplementary material for: High-resolution comparative mapping among man, cattle and mouse suggests a role for repeat sequences in mammalian genome evolution
Source: BMC Genomics. 2006 Aug 1;7:194. doi: 10.1186/1471-2164-7-194 (PMC3225868; doi:10.1186/1471-2164-7-194)
Supplement: Additional File 2 — Cattlemap_supp. This file contains a graphical version of the bovine map. [file 1471-2164-7-194-S2.doc]

34742135

21 q22.1

14593745

21q11

76747074

3 p12.3

126434359

3 q21.2

198997735

3 q27.3

132105417

3 q22.1

39945074

21 q22.3

46240082

21 q22.3

35081974

21 q22.12

39051684

21 q22.2

15271366

3 p24.3

19037639

3 p24.3

Chr3

Chr21


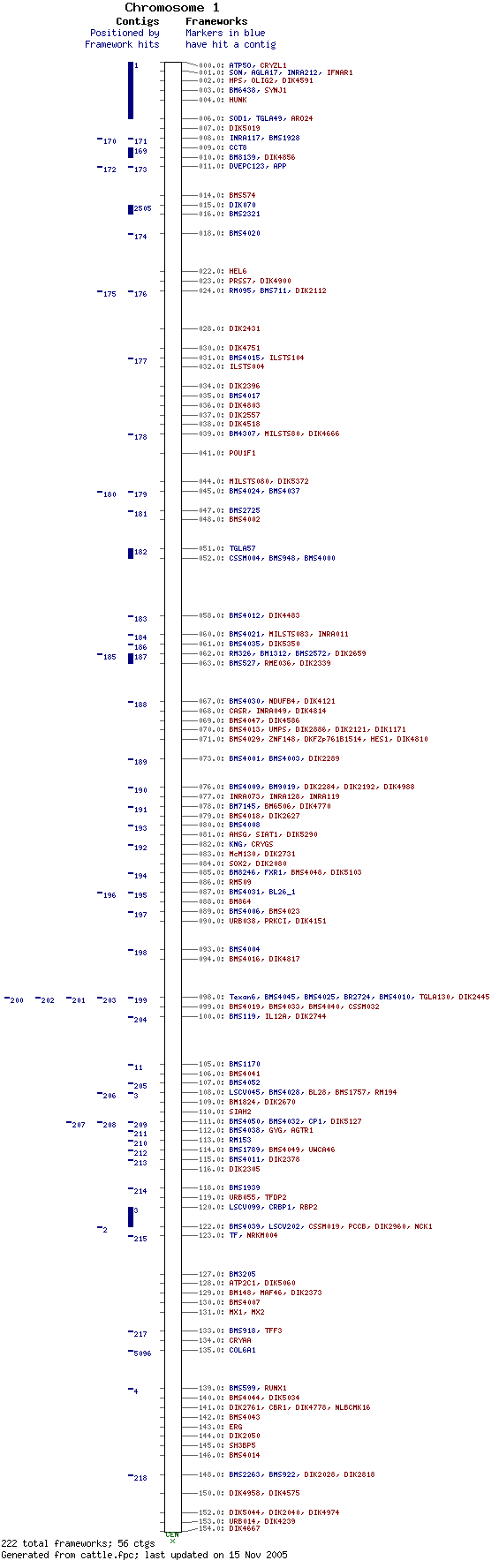


34742135

21 q22.1

14593745

21q11

76747074

3 p12.3

126434359

3 q21.2

198997735

3 q27.3

132105417

3 q22.1

39945074

21 q22.3

46240082

21 q22.3

35081974

21 q22.12

39051684

21 q22.2

15271366

3 p24.3

19037639

3 p24.3

Chr3

Chr21


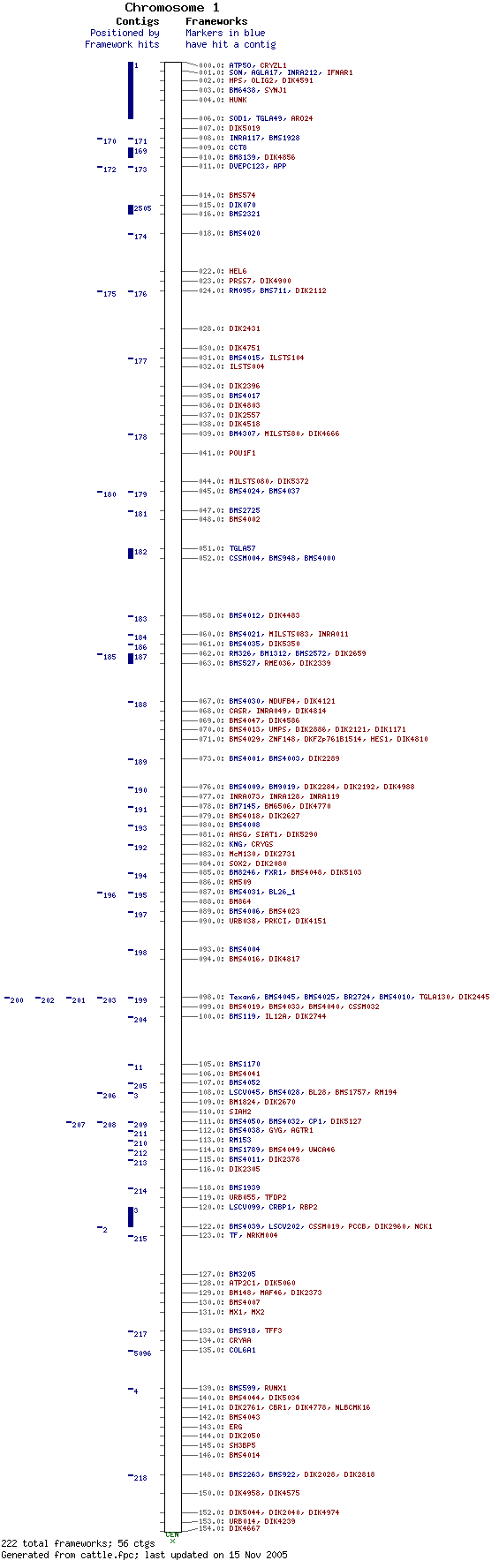


##

##


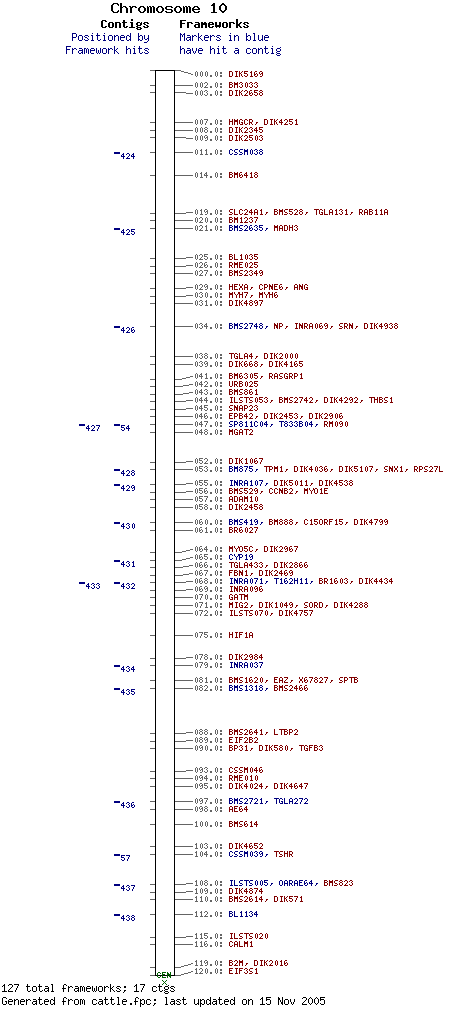


113287049

174522268

747224795 q 13.3

77647406

63632236

15 q 22.31

7035159715 q 23

2361058914 q 11.2

1892882114 q 11.2

31571124

41205484

45110659

14 q 21.3

5137781114 q 21.2

6272682115 q 22.31

4310264315 q 21.1

52393742

9056884514 q 32.11

4262381015 q 15.3

Chr5 Chr14

Chr15

##

##

##

## Bovine - human comparative map

Bovine chromosomes are indicated by vertical grey bars and markers are placed according to their framework position. Human orthologous conserved segments are presented by solid bars with one color for each human chromosome. When known, their orientation is indicated by black arrows. Human genome sequence coordinates (NCBI 35) and FISH positions of these conserved segments are shown on the right side of the conserved segments.
